# On Electricity as the Principle Which Causes the Vitality and Coafulating Property of the Blood

**Published:** 1864-04

**Authors:** R. C. Shettle

**Affiliations:** Shaftsbury


On Electricity as the Principle which causes the Vitality and Coafulating Property of the Blood. *By R. C. Shettle*, M. D., *Shaftsbury.*—The very great interest which is felt with regard to any facts bearing upon the coagulation of the blood must be my excuse for laying the results of my rough experiments before the consideration of my personal brethren. Prior to the highly interesting Croonian Lecture delivered by Mr. Lister, and lately published in The Lancet, the subject appeared to be as obscure as ever. Mr. Lister has, however, in this lecture given us very valuable information on the subject of the coagulation of the blood, not only in refuting various theories which from time to time have been advanced, but also in stating that "the real cause of the coagulation of the blood when shed from the body, is the influence exerted upon it by ordinary matter, the contact of which for a very *brief period* effects a change in the blood, inducing a mutual reaction between its solids and fluid constituents, in which the *corpuscles impart to the liquor sanguinis a disposition to coagulate.*"

For the last two years and a half I have occupied some portion of my leisure time in conducting various experiments, with a view to prove that Mr. Hunter was right in his assertion "*that the coagulation of the blood was an operation of life;*" and I have attributed its vitality to electricity. I have already communicated to The Lancet the fact that I

have detected electricity in arterial blood, and converted albumen into fibrin by means of that agent.

The following experiment, which is to a certain extent similar to some others previously undertaken, was conducted on the 13th inst, and may be relied upon as being at least accurate in not overstating facts; but the galvanometer I used, and which was one of Du Bois Reymond's, is not, I believe, so delicate as it ought to be.

*Experiment.*—The jugular vein of a dog (under the influence of chloroform), having been secured by means of two ligatures, was divided, and a glass tube inserted into the upper portion. Blood was then drawn into a clean glass vessel, taking care to avoid exposure to air as much as possible. A cork, in which was placed two platinum electrodes, covered with folds of blotting-paper and bladder, and upon which electrodes some pains had been bestowed to render them homogeneous, was then accurately fitted into the mouth of the vessel, and the wires of the electrodes applied at once to the galvanometer. The same alteration of the needle immediately followed that had succeeded the immersion of the plates into water—viz., a divergence of 45 deg. to the negative, the needle falling back again directly by zero, where, after a slight vibration or two, it remained, showing as far as the galvanometer was capable, the absence of electricity in the fluid tested.

The carotid artery of the same dog was then tied, and treated in the same way as the vein, blood being drawn into a glass vessel similar to that used for the venous blood. Electrodes in every respect precisely similar, and which also had been tested immediately before using, were inserted as before, and the wires again attached to the galvanometer. Thirty degrees of positive electricity were at once shown, and the needle was two hours and fifteen minutes returning to zero, the backward motion being very regular. The electrodes were allowed to remain in the arterial blood six hours before being again examined, when a perfectly distinct line marked the depth to which the electrodes had been immersed, the upper portion being of a very dark venous, and lower portion remaining of bright arterial hue. The clot was then removed, and found to be almost black, and not firmly coagulated.

I believe this experiment is sufficient to show that the abstraction of electricity from the blood causes it to lose its arterial character, certainly with regard to color, and, if so, must it not also be attended with loss of its stimulating property?

In a previous experiment, blood was drawn from the vein and artery of a dog, likewise under the influence of chloroform, into clean vessels. Galvanic circuits, consisting of clean plates of copper and zinc, were introduced through corks, which were accurately adjusted to the mouths of the vessels so as to exclude air. Coagulation in each instance proceeded *round the plates, especially the zincode.* On the second day a peculiar formation appeared proceeding from the zincode to the side of the vessel, which might be compared not inaptly to the growth of a fibrous clot. This formation in the venous blood was loose, and the cellular spaces between the fibres large; but in the arterial, the growth was firm in texture and smaller in size as compared with the venous. This process was allowed to go on for four days, when it had apparently ceased to increase. The vessels were then carefully opened; but owing to the plates being attached to the corks, this could not be done without disturbing the formation above alluded to, and which at once became broken up, and sank to the bottom of the vessel, presenting under the microscope granular cells. The clot, in both instances occupied not only the space between the plates, but also encrusted the zinc plate with a very firm coagulum; while on the side of the copper plate most distant from the zinc, it was scarcely to be called a fibrous clot, having more the appearance of transparent jelly. The clot taken from the zinc pole in the arterial blood had become as hard as a piece of dried leather, and, after exposure to the air, could be broken. That the coagulation of the blood is due to the action of a current of electricity, can not, I think, be reasonably doubted, inasmuch as I have shown that electricity does exist in arterial blood; and although I have not been able to detect it in venous blood, it is possible that it may exist in very small proportion, and so account for the very feeble coagulation of venous blood when perfectly excluded from the air.

The experiments of Mr. Lister, as well as those above alluded to, show that metal wires or plates, have great influence in accelerating the process of coagulation, and the influence that metals exert in attracting electricity is too well known to require comment here. That neither the coagulation nor change in color was due to chemical action, may be inferred from the fact that in the first experiment I have given the plates were platinum, covered with four folds of thick white blotting-paper which again was encased in bladder.

I would now draw attention to the mode in which I believe this electricity is generated and conveyed into the blood. Professor Faraday has well shown, and I believe it is now very generally admitted, that chemical action can not take place without the development of electricity, and indeed that the amount of electricity corresponds exactly with the amount of chemical action which has been set up. If such be the case, the amount of electricity generated in the lungs under the chemical changes which take place, there must be considerable; and we have no difficulty whatever in obtaining the amount required, neither can we remain in ignorance as to the direction such electricity must travel, when we remember one of the fundamental principles of electricity is that it must always be attracted by the best conducting medium. Now, each blood globule as it is presented in the pulmonary capillary bears with it, in the *iron* it contains, one of the best attracting agents known. It is to be presumed, consequently, that each globule *must pass away from the lungs charged with a due amount of this stimulating principle*, the immediate effect of which upon the globules would be to corrugate or contract their sides, causing them to reflect light differently—to which change in shape of the globule, change in color has latterly been attributed. Taking this view of the case, we see how very important an office the iron of the blood is appointed to serve in bearing away to all parts of the body this vitalizing principle.

DuBois Reymond, Matteucci, Aldini, &c., and last, though not least, Dr. C. B. Radcliffe, have testified to the electric current in the nerves and muscles, and all speak of the influence that arterial blood exerts over muscular contraction, &c. With a knowledge of the above facts, I think we can not do less than regard the blood as the means of supplying electricity, or, in other words, vital energy or nervous power to that great electric organ, the brain, and the whole nervous system, which may not inaptly be compared to the system of electro-telegraphy of the present day, seeing that it has been pretty clearly proved by physiologists that the nerve fibres pass from their origin in the grey substance to their peripheral extremity distinct from each other, and any njury to such fibre is followed by corresponding loss of power.

